# Synthesis and Antimicrobial Activity of some Tetrahydro Quinolone Diones and Pyrano[2,3-*d*]pyrimidine Derivatives

**Published:** 2015

**Authors:** Masoume Shahi, Naser Foroughifar, Akbar Mobinikhaledi

**Affiliations:** a *Department of Chemistry, Tehran North Branch, Islamic Azad University, Tehran, Iran.*; b *Department of Chemistry, Faculty of Science, Arak University, , 38156-8-8349, Arak, Iran.*

**Keywords:** Pyrimidine, Quinolone, Antimicrobial activity

## Abstract

There has been special interest in the chemistry of quinolone and pyrimidine derivatives due to their diverse biological activities such as anticonvulsant, anti-malarial agents, antibacterial, antiviral, cytostatic, antithelemintic, antigenotoxic, anti-cancer agents. These compounds are also used as targeting delayed-type hypersensivity and anti-convulsant agents. As a part of our research works in the synthesis of pyrimidine derivatives containing biological activities, a series of novel pyrano[2,3-*d*]pyrimidine derivatives 2 and tetrahydro quinolone dione derivatives 3 were synthesized via reaction of tetrahydrobenzo[*b*]pyrano derivatives 1 with different reagents in suitable yields. The characterization of these synthesized compounds was established by IR, ^1^H NMR and^ 13^C NMR spectroscopic data. Furthermore, all compounds were subsequently evaluated for their *in-vitro* antibacterial activity against three bacteria:* Staphylococcus aureus* (ATTC-25923), *Escherichia Coli* (ATTC-25922) and *Bacillus anthracic* (ATTC-25924).

## Introduction

Pyran derivatives are known as prevalent structural subunits in a variety of important natural products including alkaloids, carbohydrates, polyether antibiotics, pheromones, and iridoids (1). Also, compounds containing these ring systems possess a wide range of pharmacological properties such as antibacterial (2), antigenotoxic (3), antioxidant (4) and cytotoxic activity (5). On the other hand, heterocyclic compounds containing a pyrimidine or quinoline nucleus are of special interests due to their applications in medicinal chemistry as they are the basic skeleton of a number of several bioactive compounds such as antifungal (6), antibacterial (7, 8), antitumor (9), antitubercular (10, 11), anticonvulsant (12) and *ureas inhibitor (*13*)*. A combination of these two ring systems may have a variety of structural and biological activities. Therefore, preparation of heterocyclic compounds containing a pyran and quinoline moieties is still a significant synthetic challenge.

In view of these reports and also due to continuation of our works on synthesis of pyrimidines (14-17), we have developed synthesis of some novel pyrano[2,3-*d*]pyrimidine derivatives and tetrahydro quinolone dione derivatives with the hope to improve their biological activities against some gram-positive and gram-negative microorganisms.

## Experimental

 All melting points were uncorrected and measured using capillary tubes on an Electrothermal digital apparatus. IR spectra were recorded on a Shimadzo(FT)-IR 300 spectrophotometer in KBr. NMR spectra were recorded on a Brucker 500 and 300 MHz spectrometer in CDCl_3_ with TMS as an internal standard. The progress of the reaction was monitored by thin-layer chromatography TLC (Thin-Layer Chromatography) using CH_2_Cl_2_/EtOAc (3:1) as an eluent. The starting material tetrahydrobenzo[*b*]pyrano drivatives 1(a-h) are easily obtained via one pot reaction of malonitrile, dimedone and aromatic aldehyde in presence of Alum (18).

*General procedure for synthesis of pyrano[2,3-d]pyrimidine derivatives* *2(a-h)*

A solution of compound 1 (1 mmol) in Ac_2_O (1.5 mL) with catalytic amount of concentrated sulfuric acid (3-4 drops) was heated under reflux for 1 h. The reaction mixture was cooled at room temperature and kept for one day. The mixture was poured into water and the formed solid was filtrated, washed with water, and recrystallized from 2-propanol. 

*2,8,8-Trimethyl-5-phenyl-5,7,8,9-tetrahydro-4H-chromno-[2,3-d]pyrimidine-4,6(3H)-dione (2a)*

White solid; m.p. 256-258 ^o^C; Yield 60%; IR (KBr) ν_max_ (cm^-1^): 3400 (NH), 2962 (CH), 1674, 1610 (C=O) and1452 (C=N). ^1^H NMR (CDCl_3_) δ ppm: 1.05, 1.12 (both s, 3H each, C(8) (CH_3_)_2_); 2.35 (s , 3H, C(2)-CH_3_); 2.26 (m, 2H, CH_2_); 2.58 (m. 2H, CH_2_); 4.92 (s, 1H, H-5)); 7.12-7.32 (m, 5H, C_6_H_5_) and 13.10 (s, 1H, NH). ^13^C NMR (CDCl_3_) δ ppm: 21.30, 27.74, 29.29, 32.51, 33.28, 41.12, 50.89, 103.02, 114.50, 127.02, 128.29, 128.66, 128.29, 143.32, 148.31, 158.56, 161.15, 165.44 and 196.62. 

*2,8,8-Trimethyl-5-(4-methylphenyl)-5,7,8,9-tetrahydro-4H-chromno-[2,3-d]pyrimidine-4,6(3H)-dione (*2b*)*

 White solid; m.p. 238-239 ^o^C; Yield 50%; IR (KBr) ν_max_ (cm^-1^): 3430 (NH), 2961 (CH), 1670, 1610 (C=O) and 1512 (C=N). ^1^H NMR (CDCl_3_) δ ppm: 1.05, 1.11 (both s, 3H each, C(8) (CH_3_)_2_); 2.24, 2.37 (both s, 3H each, C(5)-p-CH_3_-Phenyl, C(2)-CH_3_);; 2.28 (m, 2H, CH_2_); 2.57 (m, 2H, CH_2_); 4.88 (s, 1H, H(5)); 7.00-7.11 (m, 4H, Ar-H) and 13.10 (br, 1H, NH).^ 13^C NMR (CDCl_3_) δ ppm: 21.40, 27.77, 29.30, 32.51, 32.83, 41.12, 50.92, 103.17, 114.98, 128.49, 129.021, 136.57 140.45, 158.45, 161.05, 163.40, 165.29 and 196.66. 

*2,8,8-Trimethyl-5-(3-nitrophenyl)-5,7,8,9-tetrahydro-4H-chromno-[2,3-d]pyrimidine-4,6(3H)-dione (2c)*

 Pale Yellow solid; m.p>285^o^C; Yield 81%; IR (KBr) ν_max_ (cm^-1^): 3439(NH), 2961 (CH), 1674, 1632 (C=O) and 1526 (C=N). ^1^H NMR (CDCl_3_) δ ppm: 1.10, 1.16 (both s, 3H each, C(8) (CH_3_)_2_); 2.26 (s, 3H, C(2)-CH_3_); 2.40 (m, 2H, CH_2_); 2.65 (m, 2H, CH_2_); 5.04 (s, 1H, H(5)); 7.40-8.21 (m, 4H, Ar-H) and 13.35 (br, 1H, NH).^13^C NMR (CDCl_3_) δ ppm: 21.46, 27.76, 29.23, 32.56, 33.52, 41.10, 50.77, 101.74, 113.72, 122.16, 123.81, 129.07, 134.80, 145.33, 148.29, 159.36, 161.27 165.27 and 195.53. 

*2,8,8-Trimethyl-5-(2-chlorophenyl)-5,7,8,9-tetrahydro-4H-chromno-[2,3-d]pyrimidine-4,6(3H)-dione (2d)*

White solid; m.p. 224-225 ^o^C; Yield 50%; IR (KBr) ν_max_ (cm^-1^): 3430 (NH), 2961 (CH), 1663, 1620 (C=O) and 1512 (C=N). ^1^H NMR (CDCl_3_) δ ppm: 1.07, 1.15 (both s, 3H each, C(8) (CH_3_)_2_); 2.21(m, 2H, CH_2_); 2.50(s, 3H, C(2)-CH_3_); 2.57 (m, 2H, CH_2_); 5.05 (s, 1H, H(5)); 7.01-7.50 (m, 4H, Ar-H) and 13.10 (br, 1H, NH). ^13^C NMR (CDCl_3_) δ ppm: 27.40, 29.52, 32.05, 32.25, 41.70, 40.09, 50.87, 113.87, 115.43, 126.56, 126.90, 127.91, 130.00, 130.37, 131.83, 133.12, 133.63, 140.06, 161.27 163.27 and 196.84.

*2,8,8-Trimethyl-5-(4-nitrophenyl)-5,7,8,9-tetrahydro-4H-chromno-[2,3-d]pyrimidine-4,6(3H)-dione (2e)*

 White solid; m.p. 250-251 ^o^C; Yield 70%; IR (KBr) ν_max_ (cm^-1^): 3438 (NH), 2926 (CH), 1655, 1610 (C=O) and 1510 (C=N). ^1^H NMR (CDCl_3_) δ ppm: 1.05, 1.14 (both s, 3H each, C(8) (CH_3_)_2_); 2.31(m, 2H, CH_2_); 2.40 (s, 3H, C(2)-CH_3_); 2.61 (m, 2H, CH_2_); 5.02 (s, 1H, H(5)); 8.11-7.51 (m, 4H, Ar-H) and 13.10 (br, 1H, NH).

*2,8,8-Trimethyl-5-(4-bromophenyl)-5,7,8,9-tetrahydro-4H-chromno-[2,3-d]pyrimidine-4,6(3H)-dione (2f)*

Pale yellow solid; m.p. >310 ^o^C; Yield 51%; IR (KBr) ν_max_ (cm^-1^): 3431 (NH), 2959 (CH), 1667, 1611 (C=O) and 1485 (C=N). ^1^H NMR (CDCl_3_) δ ppm: 1.05, 1.13 (both s, 3H each, C(8) (CH_3_)_2_); 2.23 (m, 2H, CH_2_); 2.36 (s, 3H, C(2)-CH_3_); 2.58 (m, 2H, CH_2_); 4.88 (s, 1H, H(5)); 7.18-7.33 (m, 4H, Ar-H) and 13.10 (br, 1H, NH).

*2,8,8-Trimethyl-5-(4-methoxyphenyl)-5,7,8,9-tetrahydro-4H-chromno-[2,3-d]pyrimidine-4,6(3H)-dione (2g)*

Cream solid; m.p. 220-221 ^o^C; Yield 60%; IR (KBr) ν_max_ (cm^-1^): 3457 (NH), 2930 (CH), 1659, 1640 (C=O) and 1504 (C=N).^ 1^H NMR (CDCl_3_) δ ppm: 1.11, 1.18 (both s, 3H each, C(8) (CH_3_)_2_); 2.25 (m, 2H, CH_2_); 2.33 (s, 3H, C(2)-CH_3_); 2.59 (m, 2H, CH_2_); 3.68 (s, 3H, O-CH_3_); 4.68 (s, 1H, H(5)); 7.07-7.11 (m, 4H, Ar-H) and 13.03 (br, 1H, NH).

*2,8,8-Trimethyl-5-(3-hydroxyphenyl)-5,7,8,9-tetrahydro-4H-chromno-[2,3-d]pyrimidine-4,6(3H)-dione* *(2h)*

White solid; m.p. 201-203 ^o^C; Yield 67%; IR (KBr) ν_max_ (cm^-1^): 3450 (NH), 2961 (CH), 1678, 1636 (C=O) and 1488 (C=N). ^1^H NMR (CDCl_3_) δ ppm: 1.06, 1.12 (both s, 3H each, C(8) (CH_3_)_2_); 2.24 (m, 2H, CH_2_); 2.36 (s, 3H, C(2)-CH_3_); 2.59 (m, 2H, CH_2_); 4.94 (s, 1H, H(5)); 6.68-7.27 (m, 4H, Ar-H); 7.02 (s, 1H, OH) and 13.30 (br, 1H, NH).

*General procedure for synthesis of tetrahydro quinolone dione derivatives 3(a-g)*

Compound 1 (1 mmol) was refluxed in a mixture of hydrochloric acid (1 mL) and acetic acid (3mL) for 3-5 h (monitored by TLC). After completion of the reaction, the reaction mixture was cooled, poured into water and the formed solid was filtrated. The obtained solid product was washed with water (3×15 mL) and recrystallized from ethanol.

*3,4,7,8-Tetrahydro-7-7-dimethyl-4-phenyl-quinoline-2,5(1H,6H)-dione (*3a*)*

 White solid; m.p. 169-171 ^o^C; Yield 48%; IR (KBr) ν_max_ (cm^-1^): 3235 (NH), 2946 (CH), 1716, 1612 (C=O). ^1^H NMR (CDCl_3_) δ ppm: 1.07, 1.18 (both s, 3H each, C (7) (CH_3_)_2_); 2.33 (m, 2H, CH_2_); 2.49 (m, 2H, CH_2_); 2.81 (m. 2H, CH_2_); 4.38 (d, 1H, H (4)); 7.29 (m, 5H, C_6_H_5_) and 8.42 (s, 1H, NH). ^13^C NMR (CDCl_3_) δ ppm: 27.88, 29.25, 33.02, 33.94, 38.10, 41.07, 46.67, 50.79, 114.84, 126.83, 127.17, 129.01, 130.06, 142.22, 150.97, 172.87 and 196.10. 

*3,4,7,8-Tetrahydro-7-7-dimethyl-4-(4-methylphenyl)-quinoline-2,5(1H,6H)-dione (3b)*

 White solid; m.p. 201-203 ^o^C; Yield 67%; IR (KBr) ν_max_ (cm^-1^): 3219 (NH), 2960 (CH), 1695, 1645 (C=O). ^1^H NMR (CDCl_3_) δ ppm: 0.92, 1.03 (both s, 3H each, C (7) (CH_3_)_2_); 2.21 (s, 3H, C(4)-*p*-CH_3_-Phenyl); 2.27 (m, 2H, CH_2_); 2.39 (m, 2H, CH_2_); 2.87(m. 2H, CH_2_); 4.31(d, 1H, H(4)); 7.03-7.25 (m, 4H, C_6_H_5_) and 8.80 (s, 1H, NH).^ 13^C NMR (CDCl_3_) δ ppm: 27.79, 29.04, 33.42, 32.83, 37.97, 41.12, 50.71, 115.03, 126.05, 129.47, 136.48, 139.12, 150.13, 172.23 and 195.49.

*3,4,7,8-Tetrahydro-7-7-dimethyl-4-(3-Nitrophenyl)-quinoline-2,5(1H,6H)-dione (3c)*

Pale Yellow solid; m.p. 194-195 ^o^C; Yield 90%; IR (KBr) ν_max_ (cm^-1^): 3105 (NH), 2960 (CH), 1707, 1620 (C=O). ^1^H NMR (CDCl_3_) δ ppm: 1.12, 1.80 (both s, 3H each, C(7) (CH_3_)_2_); 2.36 (m, 2H, CH_2_); 2.47 (m, 2H, CH_2_); 2.68 (m, 2H, CH_2_); 4.38 (d, 1H, H(4)); 7.60-8.09 (m, 4H, Ar-H) and 8.36 (s, 1H, NH).^ 13^C NMR (CDCl_3_) δ ppm: 27.80, 29.30, 33.08, 33.97, 37.80, 41.29, 50.69, 113.68, 121.64, 122.41, 130.09, 133.61, 151.39, 171.31and 195.52.

*3,4,7,8-Tetrahydro-7-7-dimethyl-4-(2-chlorophenyl)-quinoline-2,5(1H,6H)-dione (3d)*

White solid; m.p. 240-241 ^o^C; Yield 63%; IR (KBr) νmax (cm^-1^): 3247 (NH), 2961 (CH), 1715, 1645 (C=O). ^1^H NMR (CDCl_3_) δ ppm: 1.08, 1.18 (both s, 3H each, C (7) (CH_3_)_2_); 2.39 (m, 2H, CH_2_); 2.53 (m, 2H, CH_2_); 2.81 (m, 2H, CH_2_); 4.38 (d, 1H, H(4)); 7.47-8.11 (m, 4H, Ar-H) and 8.42 (s, 1H, NH).

*3,4,7,8-Tetrahydro-7-7-dimethyl-4-(4-Nitrophenyl)-quinoline-2,5(1H,6H)-dione (3e)*

White solid; m.p. 214-215 ^o^C; Yield 55%; IR (KBr) ν_max_ (cm^-1^): 3250 (NH), 2964 (CH), 1710, 1610 (C=O). ^1^H NMR (CDCl_3_) δ ppm: 1.06, 1.14 (both s, 3H each, C (7) (CH_3_)_2_); 2.32 (m, 2H, CH_2_); 2.45 (m, 2H, CH_2_); 2.90 (m, 2H, CH_2_); 4.31 (d, 1H, H (4)); 6.70-6.82 (m, 4H, Ar-H) and 8.32 (s, 1H, NH).

*3,4,7,8-Tetrahydro-7-7-dimethyl-4-(4-bromophenyl)-quinoline-2,5(1H,6H)-dione (3f)*

White solid; m.p. 173-174 ^o^C; Yield 86%; IR (KBr) ν_max_ (cm^-1^): 3208 (NH), 2945 (CH), 1667, 1636 (C=O). ^1^H NMR (CDCl_3_) (CDCl_3_) δ ppm: 1.11, 1.14 (both s, 3H each, C (7) (CH_3_)_2_); 2.31 (m, 2H, CH_2_); 2.47 (m, 2H, CH_2_); 2.92 (m, 2H, CH_2_); 4.32 (d, 1H, H (4)) and 7.10-7.40 (m, 4H, Ar-H).

*3,4,7,8-Tetrahydro-7-7-dimethyl-4-(4-methoxyphenyl)-quinoline-2,5(1H,6H) dione (3g)*

Pale yellow solid; m.p. 246-247 ^o^C; Yield 67%; IR (KBr) ν_max_ (cm^-1^): 3315 (NH), 2953 (CH), 1663, 1624 (C=O). ^1^H NMR (CDCl_3_) (CDCl_3_) δ ppm: 1.04, 1.13 (both s, 3H each, C(7) (CH_3_)_2_); 2.15 (m, 2H, CH_2_); 2.46 (m, 2H, CH_2_); 2.90 (m, 2H, CH_2_); 3.73 (s, 3H, O-CH_3_); 4.70 (d, 1H, H(4)); 6.75-7.22 (m, 4H, Ar-H) and 8.32 (s, 1H, NH).

*Antibacterial activity*

Antibacterial activity of synthesized compounds was assessed by the disc diffusion method (19) using Mueller–Hinton Agar against *Escherichia Coli* (ATTC-25922) as a gram negative bacteria as well as *Bacillus anthracic* (ATTC-25924) and *Staphylococcus aureus* (ATTC-25923) as gram positive bacteria. Cefazolin was used as a standard. Normal saline was used for preparation of inoculants having turbidity equal to 0.5 McFarland standards. The compounds were dissolved in dimethylformamide (DMF) for bioassay. The solvent control was included, although no inhibition zone was found. The plates were incubated at 37 C for 24 h. All samples were tested in triplicate and the average results of inhibitory effects are illustrated in Table 1.

**Table 1:** Antibacterial activity of newly synthesized compounds (inhibition zones, mm).

Comp. No	*E. Coli*	*Ba. anthracic*	*St. aureus*
**2a**	-	15	10
**2b**	11	15	17
**2c**	-	14	20
**2d**	13	10	3
**2e**	18	14	5
**2f**	15	18	4
**2g**	16	15	10
**2h**	12	10	10
**3a**	18	17	23
**3b**	10	15	7
**3c**	10	11	17
**3d**	10	15	10
**3e**	14	15	3
**3f**	13	10	17
**3g**	12	10	7
***Cefazolin***	13	8	6

Determination of the minimum inhibitory concentration (MIC) values for synthesized compounds against three microorganisms was carried out using disc diffusion method (20). In this method, concentrations of 1800, 900, 450, 225, 112.5, 56.2, 28.1, 14, 7, 3.5, 1.7 and 0.87 μg mL^-1 ^were used per disc and incubated at 37 ºC for 24 h.

Values of minimum inhibitor concentration (MIC) were recorded as the lowest concentration of substance, which gives no growth of inoculated bacteria. The Results are presented in Table 2.

**Table 2:** MIC values of compounds 2(a-h) and 3(a-g).

Comp. No	MIC (μg.mL^-1^)		
	*E. Coli*	*Ba. anthracic*	*St. aureus*
**2a**	225	450	112
**2b**	NP	1800	1800
**2c**	900	900	112
**2d**	225	450	112
**2e**	450	1800	900
**2f**	450	1800	900
**2g**	225	900	112
**2h**	1800	NP	1800
**3a**	900	NP	112
**3b**	450	112	450
**3c**	900	NP	1800
**3d**	900	NP	1800
**3e**	450	900	450
**3f**	450	1800	900
**3g**	NP	NP	NP
***Cefazolin***	450	900	NP

*NP*: not performed

## Results and Discussion

**Scheme 1:**
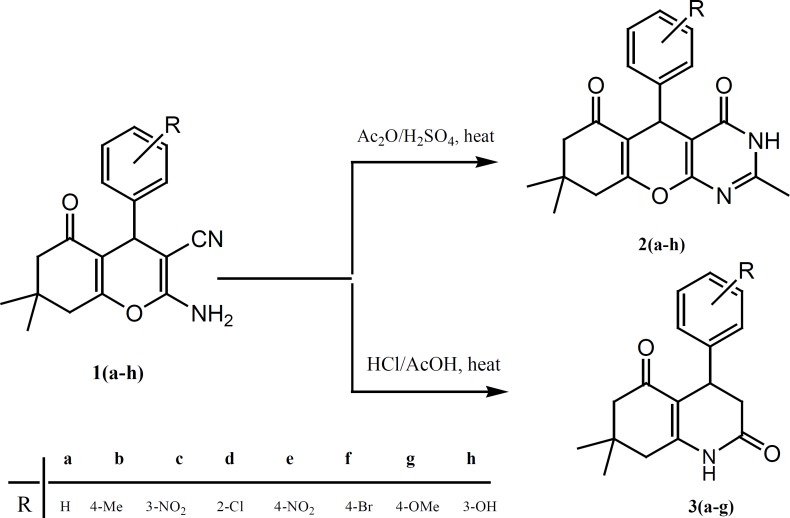
The synthetic pathway for preparation of pyrano[2,3-d]pyrimidine derivatives 2(a-h) and tetrahydro quinolone dione derivatives 3(a-g).

**Scheme 2:**
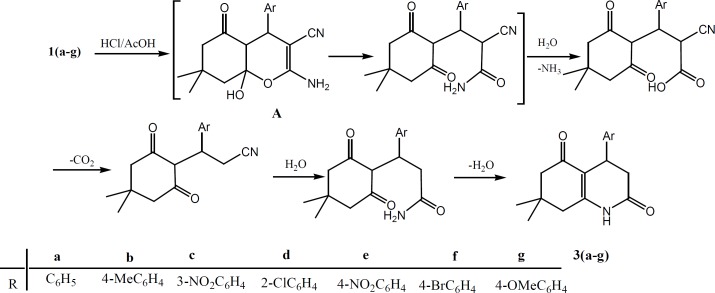
The possible mechanism for formation of compounds 3(a-g).

Compounds 1(a-h) were used as precursors for the synthesizes of pyrano[2,3-*d*]pyrimidine derivatives 2(a-h) and tetrahydro quinolone dione derivatives 3(a-g), scheme 1. The reaction of compounds 1(a-h) with a mixture of acetic anhydride in the presence of sulfuric acid under reflux, produced pyrano[2,3-*d*]pyrimidine derivatives 2(a-h), which is similar to reaction reported in the literature (21). However, different transformations occurred when refluxing of compounds 1(a-g) in concentration hydrochloric acid and acetic acid was carried out to give tetrahydro quinolone dione derivatives 3(a-g). The possible mechanism is shown in scheme 2. Compound 1 under acidic condition gives intermediate A, which can undergo a ring opening to produce an amide. The hydrolysis of amide makes an acid, followed by the loss of CO_2_, hydrolysis of CN group, and ring closure to give the more stable compound 3. 

In the IR spectra of compound 1 the nitrile and amine groups were observed in the region of 2190 and 3400 cm^-1^ (17), whereas these bands are absent in the IR spectra of compounds 2 and 3. The broad absorption band for stretching vibration of NH group was detected in the region of 3200-3450 cm^-1^, which corresponds to the pyrimidine fragment with strong hydrogen bonds. The appearance of absorption bands at 1663-1710 cm^-1^ and 1610-1645 cm^-1^ are the characteristics of the ketone and amide carbonyl groups, respectively. In ^1^H NMR spectra of these compounds the resonance of NH proton with one integration for pyrimidine ring (compounds 2) and amid group (compound 3) was observed in the region of 13.0 and 8.3 ppm, which is in support of these transformations. The resonance of all other protons appeared in the expected region of spectra. In ^13^C NMR spectra of compound 3, the appearance of two signals at about 172 and 195 ppm are due to carbon resonance of two carbonyl groups.

All synthesized compounds were tested for their antimicrobial activity by minimum inhibitory concentration (MIC) *in-vitro* by agar micro dilution method. The results were summarized in Tables 1 and 2. As depicted in Table 1, the most of the synthesized compounds proved to be effective antibacterial against three tested microorganisms, except for 2a and 2c, which were inactive against *E. Coli*. Compound 3a, showed the highest antimicrobial activity against all bacteria in general, while compounds 2d, 2e, 2f, and 3e showed the lowest activity against *St. aureus*. The other compounds exerted moderate to good activity against all stains in comparison with *Cefazolin*.
